# Reflection on EFL/ESL Teachers' Emotional Creativity and Students L2 Engagement

**DOI:** 10.3389/fpsyg.2021.758931

**Published:** 2021-10-05

**Authors:** Xianyi Sun, Jiao Li, Lan Meng

**Affiliations:** ^1^School of Education, University of Strathclyde, Glasgow, United Kingdom; ^2^School of Education, University of Glasgow, Glasgow, United Kingdom; ^3^College of Foreign Studies, Guangxi Normal University, Guilin, China

**Keywords:** emotion, L2 engagement, EFL/ESL, emotional creativity, emotion aspect

## Abstract

Emotions are one of the pillars of all human beings which can play a vital role in providing education. Emotions can affect all aspects of education. The feeling of creativity is one of the subsets of emotions. This feeling strongly affects the performance of education and the level of involvement of students. Student involvement has different aspects: social aspect; individual aspect, and emotional aspect. The present review shows that the emotional aspect of L2 engagement plays a pivotal role in the process of learning the language in English as a foreign language (EFL) and English as a second language (ESL) context. In dealing with the emotional aspect of teachers, the personal, social, and environmental aspects of the individual should be considered. The paper concludes with some pedagogical implications and provides some suggestions for future research.

## Introduction

In the last two decades, second and foreign language researchers have focused on factors that increase or decrease learners' engagement (Aubrey et al., 2020; Zhang, 2020). Teachers in different educational environments around the world have mentioned that student engagement and lack of focus on learning are the biggest problems of most educational environments. Many of them believe that action and attention are the most important factors in creating active engagement and the existence of various distracters is the prominent hindrance in its creation (Mercer and Dörnyei, 2020). Research has shown that language learners' high engagement has many positive results such as high levels of perseverance (Dao, 2020), remarkable academic achievements (Dörnyei and Al-Hoorie, 2017; Phung, 2017), increased mental health (Dewaele et al., 2019; Ellis, 2019), reduced dropout rates (Martin et al., 2017), reduced amount of boredom (Derakhshan et al., 2021), and reduced risky behaviors in the language teaching process (Christenson et al., 2012).

Many definitions have been offered for learners' engagements (Hiver et al., 2021). However, the common features of this notion are as follow: it needs the active involvement of language learners' in pedagogical tasks (Lambert and Zhang, 2019); it is context and culture-dependent (Martin et al., 2017), intrinsically situated (Mercer and Dörnyei, 2020), and dynamic and malleable (Greenier et al., 2021; Wang and Derakhshan, 2021). Regarding Svalberg, 2017 work, engagement can be evaluated from three different processes that are cognitive, affective, and social processes. Among them, the affective dimension plays a central role in teaching and learning a new language and can affect two other dimensions (Henry, 2019). It is directly related to the psychology of language teaching (Pishghadam et al., 2021a).

One of the areas that have received special attention in the last decade is Positive Psychology (PosPsy). Contrary to the traditional psychological view, which focuses only on the existing shortcomings and the ways to reduce or eliminate them, by focusing on an ideal life, positive psychology tries to help people develop their abilities and lead them to their potential (MacIntyre and Mercer, 2014; MacIntyre et al., 2016; Dewaele et al., 2019; Wang et al., 2021). For instance, instead of focusing on Foreign Language Anxiety (FLA), language researchers in positive psychology looked at positive emotions or learners' pleasure and motivation (Fathi and Derakhshan, 2019). However, the emotions of teachers and students can be facilitating or limiting, motivating or demotivating, positive or negative. Researchers claim that teachers have the ability to control students' positive and negative emotions by using strategies such as boosting students' imaginations and relaxing practice in language classes (Talbot and Mercer, 2018). PosPsy as a scientific study seeks to help better understand the important conditions and processes in the flourishing or optimal performance of students and all people in language teaching environments. However, few researchers have examined positive psychology in language teaching field of study and its sub-disciplines (Jiang et al., 2016).

## Creativity and Emotional Creativity in ELT

Before introducing some theories and exercises about creativity in English language classes, it is worth mentioning the importance of creativity in this process. Using language is a creative act; strategies that are used to avoid language deficiencies in different situations mainly use imaginative and creative methods; and creative activities increase students' self-esteem, which has a great impact on their moods and emotions (Dewaele et al., 2019). In addition, learning a foreign language usually occurs to improve the quality of human life. Research in this area has shown that there is a direct relationship between lifelong learning and the general well-being of language learners. Learning a foreign language improves learners' social engagement. Therefore, learning a language can increase learners' positive emotions about themselves (Al-Hoorie, 2016).

Positive emotions are used as one of the main pillars of positive psychology (Henry and Thorsen, 2020; Wang et al., 2021). They are also used as a tool for achieving mental growth, educational growth, intellectual development, and improving learners' engagement. Proponents of this view consider individual creativity as a structure related to emotions and focus on a concept called emotional creativity. According to their definition, emotional creativity (EC) is a voluntary trait that encompasses all of one's emotional experiences. They believe that the environment and social norms can have a remarkable impact on the performance of individuals' emotional creativity. Considering a cognitive root for this concept, they also believe that the formation and development of this concept starts from the childhood. This new trend influences various fields of study, and second/foreign language teaching was not an exception (Lambert and Zhang, 2019).

One of the most important characteristics of emotional creativity is the ability of language learners to recognize, understand and express, organize, and use their own emotions and those of others (Mystkowska-Wiertelak, 2020). Dörnyei and Al-Hoorie (2017) stated that emotional creativity has four dimensions: novelty, effectiveness, authenticity, and preparedness. Researchers believe that emotional creativity can be employed as a predictor of learners' language creativity and language achievements. Different types of emotions affect learning processes. Different people such as teachers, learners, parents, and principals in an educational environment have or experience different emotions. Emotions in language classes can affect students' academic performance as well as their interest, commitment, and personality development. These emotions can also affect the social atmosphere of classrooms and learning environments. When pedagogical tasks evoke pleasure, joy, or hope, language learners become more motivated and more attentive before doing work (Amini Faskhodi and Siyyari, 2018). This motivation and attention will increase their engagement in classroom activities. Conversely, experiencing negative emotions may lead to poor academic achievements. Those learners who possess emotional creativity can control positive and negative emotions and lead them to deep and effective learning. Thus, the emotions that language learners experienced in their classrooms can predict the language learners' engagement level. It shows that emotions are the most prominent of every language teaching context. Thus, emotional engagement is extremely important in language classes (Derakhshan et al., 2020).

As mentioned before, in foreign language learning environments, emotional engagement occurs when learners participate in classroom activities and are influenced by the emotional reactions associated with that activity. Studies have shown that language learners have emotional engagement and a positive and purposeful attitude toward classroom activities (Skaalvik and Skaalvik, 2014). Positive emotions are expressed more in the form of pleasure, passion, and expectation. On the other hand, language learners who have not experienced the desired level of emotional engagement involve in negative emotions such as anxiety, boredom, frustration, and anger (Derakhshan et al., 2021; Wang and Derakhshan, 2021). These studies also showed that emotional engagement as one of the most important elements of class engagement has a significant impact on other dimensions of interaction.

Social engagement as another aspect of students' engagement in learning a foreign language has a special place. The social aspect of engagement is divided according to class interactions and how they are (Azkarai and Kopinska, 2020). The main purpose of this dimension after the engagement of language learners is to interact with and support other components of the learning environment (Chen and Kent, 2020). Social engagement is the foundation of connecting learners in the classroom. Research findings show that negative and positive emotions are not exclusive and often exist in the language learning process and may have a positive or negative effect on language learning in interaction with cognitive and contextual factors (Runhaar et al., 2013).

## Conclusion

Because emotions are somewhat controllable, researchers believe that teachers have the potential to influence and help students' emotions and help them reach their full potential. To this end, teachers must create a safe environment in which the impact of negative emotions is even reduced (Benesch, 2018). They can use different techniques to encourage students to use their imagination and reduce negative emotions. Teachers can encourage students to substitute soothing responses when faced with such situations to reduce the intensity of emotional responses (Bielak and Mystkowska-Wiertelak, 2020). For instance, when those freshers just enter the universities, they may suffer various anxiety in learning in the institutes of higher learning. Obviously, teaching approaches may be totally different from those adopted in the senior high schools, so they would feel anxious when they have difficulty in learning. Under such circumstances, teachers in the universities have the responsibility of helping them get rid of their anxiety and teachers should give some learning guidance whenever necessary. By doing so, teachers, for one thing, can be able to help their own students to better their learning efficacy; and teachers, for another, can establish the harmonious and rapport relationship with their students. As the theory of loving pedagogy has put it, teachers' confirmation as well as teacher-student rapport relationship can facilitate the students' learning efficacy in the long run (Wang et al., 2021).

As research has shown, it is impossible to completely eliminate negative emotions. There are positive and negative emotions in the classroom at the same time in every moment of the classroom. Teachers should try to use the power of positive emotions to communicate and increase their participation. In fact, positive emotions such as satisfaction, joy, pride, and interest in students allow them to learn the language better. Focusing on positive emotions and trying to use them allows students to reduce or eliminate negative emotions. Finally, positive emotions have a significant impact on students' resilience, stubbornness, and engagement. Teachers should consider the social and emotional aspects of engagement in creating an effective and positive learning environment by focusing on teacher-student interpersonal factors such as confirmation, credibility, stroke, care, immediacy, rapport, etc. (Pishghadam et al., 2021b; Xie and Derakhshan, 2021). Culture, context, and individual differences influence learner' engagement, so further studies are required to shed light on different aspects of it. In particular, learning will take on a different look when teaching entities are different. For example, foreign language learning will be enormously influenced by their first language. Therefore, future studies should be validated in different educational contexts.

## Author Contributions

All authors listed have made a substantial, direct and intellectual contribution to the work, and approved it for publication.

## Conflict of Interest

The authors declare that the research was conducted in the absence of any commercial or financial relationships that could be construed as a potential conflict of interest.

## Publisher's Note

All claims expressed in this article are solely those of the authors and do not necessarily represent those of their affiliated organizations, or those of the publisher, the editors and the reviewers. Any product that may be evaluated in this article, or claim that may be made by its manufacturer, is not guaranteed or endorsed by the publisher.

## References

[B1] Al-Hoorie A. H. (2016). Unconscious motivation. Part II: Implicit attitudes and L2 achievement. Stud. Second Lang. Learn. Teach. 6, 619–649. 10.14746/ssllt.2016.6.4.4

[B2] Amini Faskhodi A. Siyyari M. (2018). Dimensions of work engagement and teacher burnout: a study of relations among Iranian EFL teachers. Aust. J. Teacher Educ. 43, 78–93. 10.14221/ajte.2018v43n1.5

[B3] Aubrey S. King J. Almukhalid H. (2020). Language learner engagement during speaking tasks: a longitudinal study. RELC J. [Epub ahead of print]. 10.1177/0033688220945418

[B4] Azkarai A. Kopinska M. (2020). Young EFL learners and collaborative writing: a study on patterns of interaction, engagement in LREs, and task motivation. System 94:102338. 10.1016/j.system.2020.102338

[B5] Benesch S. (2018). Emotions as agency: feeling rules, emotion labor, and English language teachers' decision-making. System 79, 60–69. 10.1016/j.system.2018.03.015

[B6] Bielak J. Mystkowska-Wiertelak A. (2020). Language teachers' interpersonal learner-directed emotion-regulation strategies. Lang. Teach. Res. [Epub ahead of print]. 10.1177/1362168820912352

[B7] Chen J. C. C. Kent S. (2020). Task engagement, learner motivation and avatar identities of struggling English language learners in the 3D virtual world. System 88:102168. 10.1016/j.system.2019.102168

[B8] Christenson S. L. Reschly A. L. Wylie C. (Eds.) (2012). Handbook of Research on Student Engagement. New York, NY: Springer. 10.1007/978-1-4614-2018-7

[B9] Dao P. (2020). Effect of interaction strategy instruction on learner engagement in peer interaction. System 91:102244. 10.1016/j.system.2020.102244

[B10] Derakhshan A. Coombe C. Zhaleh K. Tabatabaien M. (2020). Examining the roles of professional development needs and conceptions of research in English language teachers' success. TESL-EJ 24, 1–28. Available online at: http://www.teslej.org/wordpress/issues/volume24/ej95/ej95a2/ (accessed May, 2021).

[B11] Derakhshan A. Kruk M. Mehdizadeh M. Pawlak M. (2021). Boredom in online classes in the Iranian EFL context: sources and solutions. System 101:102556. 10.1016/j.system.2021.102556

[B12] Dewaele J. M. Chen X. Padilla A. M. Lake J. (2019). The flowering of positive psychology in foreign language teaching and acquisition research. Front. Psychol. 10:2128. 10.3389/fpsyg.2019.02128 31607981 PMC6769100

[B13] Dörnyei Z. Al-Hoorie A.H. (2017). The motivational foundation of learning languages other than Global English. Modern Lang. J. 101, 455–468. 10.1111/modl.12408

[B14] Ellis N.C. (2019). Essentials of a theory of language cognition. Modern Lang. J. 103, 39–60. 10.1111/modl.12532

[B15] Fathi J. Derakhshan A. (2019). Teacher self-efficacy and emotional regulation as predictors of teaching stress: an investigation of Iranian English language teachers. Teach. Eng. Lang. 13, 117–143. 10.22132/TEL.2019.95883

[B16] Greenier V. Derakhshan A. Fathi J. (2021). Emotion regulation and psychological well-being in teacher work engagement: a case of British and Iranian English language teachers. System 97:102446. 10.1016/j.system.2020.102446

[B17] Henry A. (2019). Online media creation and L2 motivation: a socially-situated perspective. TESOL Q. 53, 372–404. 10.1002/tesq.485

[B18] Henry A. Thorsen C. (2020). Disaffection and agentic engagement: ‘Redesigning’ activities to enable authentic self-expression. Lang. Teach. Res. 24, 456–475. 10.1177/1362168818795976

[B19] Hiver P. Al-Hoorie A. H. Vitta J. P. Wu J. (2021). Engagement in language learning: a systematic review of 20 years of research methods and definitions. Lang. Teach. Res. [Epub ahead of print]. 10.1177/13621688211001289

[B20] Jiang J. Vauras M. Volet S. Wang Y. (2016). Teachers' emotions and emotion regulation strategies: self- and students' perceptions. Teach. Teach. Educ. 54, 22–31. 10.1016/j.tate.2015.11.008

[B21] Lambert C. Zhang G. (2019). Engagement in the use of English and Chinese as foreign languages: the role of learner-generated content in instructional task design. Modern Lang. J. 103, 391–411. 10.1111/modl.12560

[B22] MacIntyre P. D. Gregersen T. Mercer S. (2016). (Eds.). Positive Psychology in SLA. Bristol: Multilingual Matters. 10.21832/9781783095360

[B23] MacIntyre P. D. Mercer S. (2014). Introducing positive psychology to SLA. Stud. Second Lang. Learn. Teach. 4, 153–172. 10.14746/ssllt.2014.4.2.2

[B24] Martin A.J. Ginns P. Papworth B. (2017). Motivation and engagement: same or different? Does it matter? Learn. Indiv. Diff. 55, 150–162. 10.1016/j.lindif.2017.03.013

[B25] Mercer S. Dörnyei Z. (2020). Engaging Language Learners in Contemporary Classrooms. Cambridge: Cambridge University Press. 10.1017/9781009024563

[B26] Mystkowska-Wiertelak A. (2020). Teachers' accounts of learners' engagement and disaffection in the language classroom. Lang. Learn. J. [Epub ahead of print]. 10.1080/09571736.2020.1800067

[B27] Phung L. (2017). Task preference, affective response, and engagement in L2 use in a US university context. Lang. Teach. Res. 21, 751–766. 10.1177/1362168816683561

[B28] Pishghadam R. Derakhshan A. Jajarmi H. Tabatabaee Farani S. Shayesteh S. (2021a). Examining the role of teachers' stroking behaviors in EFL learners' active/passive motivation and teacher success. Front. Psychol. 12:3034. 10.3389/fpsyg.2021.707314 34354648 PMC8329251

[B29] Pishghadam R. Derakhshan A. Zhaleh K. Al-Obaydi L. H. (2021b). Students' willingness to attend EFL classes with respect to teachers' credibility, stroke, and success: a cross-cultural study of Iranian and Iraqi students' perceptions. Curr. Psychol. [Epub ahead of print]. 10.1007/s12144-021-01738-z

[B30] Runhaar P. Konermann J. Sanders K. (2013). Teachers' organizational citizenship behaviour: considering the roles of their work engagement, autonomy and leaderemember exchange. Teach. Teach. Educ. 30, 99–108. 10.1016/j.tate.2012.10.008

[B31] Skaalvik E. M. Skaalvik S. (2014). Teacher self-efficacy and perceived autonomy: relations with teacher engagement, job satisfaction, and emotional exhaustion. Psychol. Rep. 114, 68–77. 10.2466/14.02.PR0.114k14w0 24765710

[B32] Svalberg A. M. L. (2017). Researching language engagement; current trends and future directions. Lang. Awareness 27, 21–39. 10.1080/09658416.2017.1406490

[B33] Talbot K. Mercer S. (2018). Exploring university ESL/EFL teachers' emotional well-being and emotional regulation in the United States, Japan and Austria. Chin. J. Appl. Ling. 41, 410–432. 10.1515/cjal-2018-0031

[B34] Wang Y. Derakhshan A. Zhang L. J. (2021). Researching and practicing positive psychology in second/foreign language learning and teaching: the past, current status and future directions. Front. Psychol. 12:731721. 10.3389/fpsyg.2021.731721 34489835 PMC8417049

[B35] Wang Y. L. Derakhshan A. (2021). [Review of the book Investigating dynamic relationships among individual difference variables in learning English as a foreign language in a virtual world, by M. Kruk]. System 2021:102531. 10.1016/j.system.2021.102531

[B36] Xie F. Derakhshan A. (2021). A conceptual review of positive teacher interpersonal communication behaviors in the instructional context. Front. Psychol. 12:708490. 10.3389/fpsyg.2021.708490 34335424 PMC8319622

[B37] Zhang Z. (2020). Learner engagement and language learning: a narrative inquiry of a successful language learner. Lang. Learn. J. [Epub ahead of print]. 10.1080/09571736.2020.1786712

